# Elemental and Stable Isotope Validation of Stepwise Graphene Oxide Functionalization to GRAPHYMERE^®^

**DOI:** 10.3390/ma19153206

**Published:** 2026-07-27

**Authors:** Davide Di Rosa, Gennaro Ruggiero, Francesco Caso, Mauro Rubino, Fabio Marzaioli, Roberto Sorrentino, Fernando Zarone, Giuseppe Caso

**Affiliations:** 1 Department of Mathematics and Physics, University “Luigi Vanvitelli”, 81100 Caserta, Italy; mauro.rubino@unicampania.it (M.R.); fabio.marzaioli@unicampania.it (F.M.); giuseppe.caso@unicampania.it (G.C.); 2 Scientific Unit of Digital Dentistry, Department of Neurosciences, Reproductive and Odontostomatological Sciences, Division of Prosthodontics, University “Federico II” of Naples, 80131 Naples, Italy; roberto.sorrentino@unina.it (R.S.); zarone@unina.it (F.Z.); 3 Department of Chemical Sciences, University “Federico II” of Naples, 80126 Naples, Italy; francesco.caso511@outlook.it

**Keywords:** graphene oxide, Isotope ratio mass spectrometry, stable isotopes, elemental analysis, dental materials, methacrylamide monomer, graphene functionalization, nanocomposites

## Abstract

**Highlights:**

- EA–IRMS provided complementary evidence consistent with GO functionalization.
- Stable isotope shifts revealed progressive chemical modification.
- Elemental analysis quantified nitrogen incorporation after amination.
- EA–IRMS provides bulk information complementary to FT-IR/Raman spectra.
- The approach can contribute to batch screening of complex GO derivatives.
- GRAPHYMERE^®^ was characterized through elemental and isotope descriptors.

**Abstract:**

Stepwise functionalization of graphene oxide (GO) into polymerizable derivatives requires analytical evidence able to distinguish chemical modification from the spectral overlap typical of oxidized carbon frameworks. Here, pristine GO, amine-functionalized GO (GO–ED), and the methacrylamide-modified derivative GRAPHYMERE^®^ were compared by elemental analysis and stable isotope ratio mass spectrometry. Carbon content increased progressively from GO to GRAPHYMERE^®^, while nitrogen was reproducibly incorporated after amination and retained after methacrylamide modification. The materials also showed a monotonic δ^13^C shift and distinct δ^15^N signatures for the nitrogen-containing derivatives, consistent with progressive bulk chemical modification. These elemental–isotopic trends provide complementary support for the proposed functionalization pathway and for the analytical distinction among the starting material, intermediate, and final derivative. EA–IRMS is therefore proposed as an additional batch-screening tool for chemically complex GO-based precursors intended for future polymeric and dental-material applications, without replacing bond-specific structural techniques.

## 1. Introduction

Graphene oxide (GO) is a chemically versatile graphene derivative produced by oxidative exfoliation of graphite. Its reactivity and colloidal stability are controlled by a non-uniform population of oxygen-containing functional groups (hydroxyl, epoxide, carbonyl, and carboxyl) distributed over basal planes and sheet edges. This chemical heterogeneity provides multiple reactive sites for covalent modification and supports the rational design of GO-based polymer nanocomposites [1,2].

Across many polymer matrices, GO has been investigated to modulate elastic modulus, fracture toughness, and transport/barrier performance, as well as to impart specific multifunctional properties, including thermal stabilization, anti-corrosion performance, electrical or dielectric modulation, and antibacterial or antibiofilm activity, depending on the polymer matrix, filler loading, dispersion quality, and surface chemistry [3,4,5,6,7,8,9].

For these reasons, covalent functionalization strategies are widely adopted to increase matrix compatibility, mitigate restacking (re-aggregation/re-stacking of GO sheets with a concomitant loss of accessible surface area, driven by π–π interactions and van der Waals forces), and generate chemically anchored interfaces [4,10]. In this context, grafting-to involves the covalent attachment of preformed polymer chains onto the GO surface, whereas grafting-from relies on surface-initiated polymerization, in which initiator functionalities are immobilized on GO and polymer chains grow directly from surface sites [10,11]. Both approaches increase the affinity of the GO surface for the organic phase, suppress aggregation, and reinforce the GO–polymer interphase, thereby improving stress transfer and reducing interfacial debonding [4,11]. Recent studies published between 2024 and 2026 further confirm that the performance of GO/polymer nanocomposites is strongly governed by the balance between surface functionalization, nanosheet dispersion, and interfacial load transfer, with covalent and non-covalent modifications representing key routes to reduce aggregation and improve matrix compatibility [5,6,7,8,9].

Alternatively, with the same objective of interfacial stabilization, GO can be functionalized with polymerizable groups: rather than attaching preformed chains or growing chains from the surface, reactive moieties are introduced that directly participate in polymerization/crosslinking, enabling covalent incorporation of GO-derived units into the network during curing [12]. Recent examples in high-performance epoxy nanocomposites and GO-based membranes also show that tailored GO functionalization can improve polymer compatibility, structural stability, and macroscopic performance, reinforcing the rationale for designing GO derivatives as polymer-integrated platforms rather than as passive fillers [7,8,9,13,14].

The transition from a physically blended filler to a chemically integrated filler is particularly relevant for dental resins, where water uptake, cyclic loading, and solvent exposure can accelerate interfacial degradation and promote filler leaching [15]. In this setting, GO-derived comonomers that can be co-polymerized within acrylic networks provide a direct route to enhanced interfacial stability by embedding functionalized GO within the crosslinked matrix through covalent bonding [16]. On this basis, we recently synthesized GRAPHYMERE^®^, a GO-based methacrylamide monomer, and reported its structural characterization by FT-IR and Raman spectroscopy [16].

Although vibrational spectroscopy is a key tool for tracking functional-group evolution, FT-IR/Raman interpretation in oxidized carbon frameworks is often not unique. GO exhibits broad, intensity-variable O–H stretching bands (strongly affected by hydrogen bonding and interlayer water), overlapping contributions in the C=O/C=C region, and a crowded fingerprint region where C–O, C–O–C, and C–N modes can be heavily convoluted. After amination and subsequent acrylic functionalization, aliphatic and amide-related vibrations may coincide with, or be obscured by, native GO bands. In parallel, Raman D/G descriptors primarily reflect defect density and sp^2^/sp^3^ content rather than the chemical identity of grafted species [16]. As a result, spectroscopic assignments may remain qualitative and can be ambiguous when functionalization is incomplete or when chemical heterogeneity persists [1,2].

These limitations motivate the use of independent and complementary, bulk, quantitative methods. Elemental (C, N) analysis constrains macroscopic composition along the functionalization sequence—most notably nitrogen incorporation after diamine grafting and subsequent amide formation—and enables stoichiometric consistency checks that cannot be derived from band intensities alone, while stable isotope ratio analysis by EA–IRMS (elemental analyzer–isotope ratio mass spectrometry) provides a second, complementary non-structural descriptor of chemical modification. Carbon isotope ratios (δ^13^C, relative to Vienna Pee Dee Belemnite—VPDB) report the net carbon mass balance and can capture the cumulative contribution of newly introduced carbon pools, whereas nitrogen isotope ratios (δ^15^N, relative to air) track incorporation of nitrogen-bearing functionalities and can reflect integrated isotopic shifts arising from the added nitrogen reservoir and any reaction-associated fractionation.

The aim of this study is to provide further evidence supporting the functionalization of graphene oxide (GO) achieved through the procedure described by Ruggiero et al. [16]. To this end, complementary analytical approaches were employed, including the determination of elemental composition (C, N) and the analysis of the stable isotopic signatures of carbon and nitrogen (δ^13^C and δ^15^N) in GO, GO–ED, and GRAPHYMERE^®^. This combined approach enables the chemical transformations occurring during the different stages of the functionalization process to be traced in a consistent and verifiable manner.

Isotopic analyses were performed to assess whether chemical functionalization induced detectable variations in the isotopic signature of the graphene oxide substrates. Although EA–IRMS is an established analytical technique, including for the stable carbon isotope characterization of graphite and for discriminating between natural and synthetic organic compounds, its use as a routine characterization tool for synthetic molecules and materials remains less common than conventional structural, spectroscopic, and surface-sensitive approaches [17,18]. In this work, EA–IRMS is therefore explored as a complementary bulk elemental–isotopic descriptor to follow chemical modification through elemental incorporation and isotopic mass-balance evolution. Beyond a generic polymer-composite perspective, this characterization is directly relevant to dentistry because resin-based dental materials are exposed to a particularly aggressive environment, including saliva, pH fluctuations, thermal changes, masticatory fatigue, toothbrushing abrasion, and biofilm accumulation. These conditions make the stability of the filler–matrix interface and the reproducibility of the polymer network clinically meaningful. Graphene-based additives have therefore been investigated in dental polymers, PMMA prosthetic materials, resin composites, adhesive systems, surface coating agents, resin-modified glass ionomer cements, and experimental cements because of their potential influence on mechanical behavior, surface properties, and antibacterial or antibiofilm performance [19,20,21,22,23,24,25,26,27].

Accordingly, the relevance of the present analytical work lies in providing complementary bulk elemental–isotopic evidence for distinguishing the GO-derived precursor before its incorporation into experimental resin formulations. This upstream characterization can support the interpretation of subsequent mechanical, biological, and aging studies, but does not predict improved performance after incorporation into a dental material. From this perspective, the present study adds a bulk elemental–isotopic level of characterization to the previously reported FT-IR/Raman assessment, with the aim of supporting the chemical distinction among pristine GO, GO–ED, and GRAPHYMERE^®^.

## 2. Materials and Methods

### 2.1. Materials

We used the following materials: Graphene oxide (GO) dry powder (Graphenea, San Sebastián, Spain; elemental composition provided by the supplier: 49–56 wt% C, 1–2 wt% H, 0–1 wt% N, 2–3 wt% S, and 41–50 wt% O). Methyl methacrylate (MMA) solution (>95% *m*/*v* MMA and <2% *m*/*v* N,N-dimethyl-p-toluidine; Lang Dental Manufacturing Company, Wheeling, IL, USA). Acetone (ACS reagent, ≥99.5%) Sigma-Aldrich (St. Louis, MO, USA). High-purity water from a Milli-Q system (Millipore, Merck KGaA, Darmstadt, Germany). Tin sample-capsules (Thermo Fisher Scientific Inc., Waltham, MA, USA). Atropine (Thermo Fisher Scientific Inc., Waltham, MA, USA). IAEA-C5, IAEA-C6, IAEA-600, IAEA-NO-3, IAEA-N-2 (International Atomic Energy Agency, Vienna International Centre, P.O. Box 100, 1400 Vienna, Austria).

Measurements were performed using an EA–IRMS system consisting of a Thermo Scientific™ FLASH 2000 CHNS/O elemental analyzer (Thermo Fisher Scientific Inc., Waltham, MA, USA) equipped with dual reactors for combustion and pyrolysis and a Thermo Scientific™ MAS 200R solid autosampler, coupled via a Thermo Scientific™ ConFlo™ IV (Thermo Fisher Scientific Inc., Waltham, MA, USA) universal interface to a Thermo Scientific™ DELTA V™ Advantage (Thermo Fisher Scientific Inc., Waltham, MA, USA) isotope ratio mass spectrometer operated in continuous-flow mode. Samples were automatically introduced into the EA, converted into simple analyte gases by the combustion reactor (carbon and nitrogen are respectively converted into CO_2_ and N_2_), which were subsequently chromatographically separated, quantified by a Thermal Conductivity Detector (TCD), and finally transferred to the IRMS; the ConFlo IV enabled reference-gas handling, automated dilution, and signal matching between sample and reference, ensuring reproducible measurement conditions; a helium 5.0 flow of 100 mL/min was used as carrier during the analysis process. Data acquisition, peak integration, and isotope-ratio processing were carried out using Thermo Scientific™ ISODAT software (ver. 3.0.94.12; Thermo Fisher Scientific Inc., Waltham, MA, USA).

### 2.2. Preparation of GO-Based Substrates

**Figure 1:**
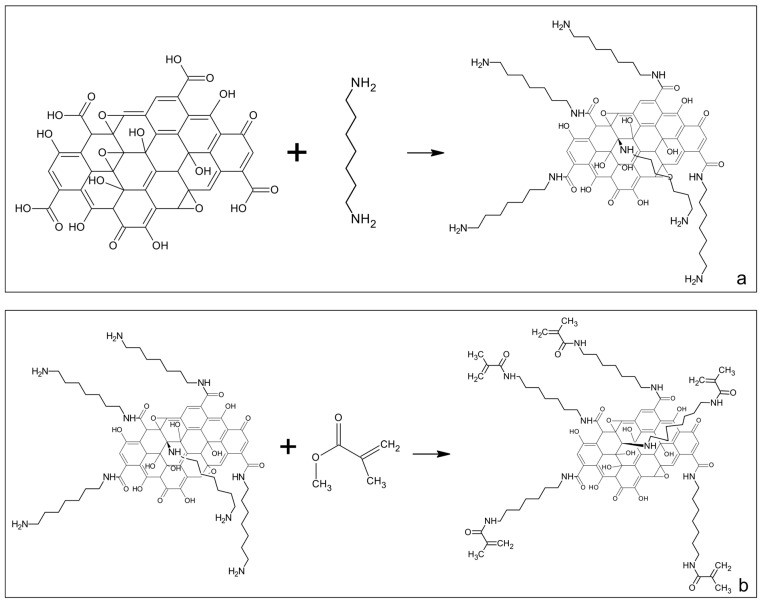
Schematic illustration of the two-step functionalization route from graphene oxide to GRAPHYMERE^®^. (**a**) Graphene oxide is functionalized with hexamethylenediamine to obtain the amine-modified intermediate GO–ED. (**b**) GO–ED is subsequently reacted with methyl methacrylate, introducing methacrylamide groups and yielding the final GRAPHYMERE^®^ monomer.

The synthesis and chemical functionalization of GO (Figure 1) were carried out following the procedure described in the article Ruggiero et al., 2025 [16]. Briefly, pristine GO was first functionalized with hexamethylenediamine to obtain an amine-modified intermediate (GO–ED). Subsequently, the amine-functionalized GO was reacted with methyl methacrylate, yielding the final graphene-oxide-based substrate (GRAPHYMERE^®^).

### 2.3. Elemental Analysis

Elemental analysis was performed to determine the carbon and nitrogen content of the three GO-based substrates (GO, GO–ED, and GRAPHYMERE^®^). Measurements were carried out on dried samples, and results are reported as weight percentages (%C and %N). Three replicates for each substrate were weighed in tin capsules and analyzed using the EA. The calibration of the EA both for the calculation of carbon and nitrogen percentage was obtained by the measurement of different aliquots of an atropine reference material.

### 2.4. Stable Isotopes Analysis

Stable isotope ratios of carbon (δ^13^C) and nitrogen (δ^15^N) were determined by isotope ratio mass spectrometry (IRMS). Isotopic compositions are reported in delta notation (δ), as shown in Equations (1) and (2):

(1) δ13CSample(‰)=RSample13RVPDB13−1×1000

(2) δ15NSample(‰)=RSample15RAIR15−1×1000

where the ^13^R_sample_ is the isotope ratio of ^13^C to ^12^C of the sample and ^13^R_VPDB_ is the isotope ratio of ^13^C to ^12^C of the Vienna Pee Dee Belemnite (VPDB) reference scale, used as international reference standard for carbon isotope ratios. Similarly, ^15^R_sample_ is the isotope ratio of ^15^N to ^14^N of the sample and ^15^R_AIR_ is the corresponding ratio of ^15^N to ^14^N of atmospheric air, which is the international reference standard for nitrogen isotope ratios. The isotope ratios (R) are dimensionless, whereas δ^13^C and δ^15^N values are conventionally reported in per mil (‰) relative to the respective international standard.

Analytical uncertainty was evaluated based on δ^13^C and δ^15^N measurements of three replicates of the GO-based substrates and is reported as one standard deviation.

δ^13^C and δ^15^N values were normalized to the international VPDB and AIR scales, respectively, using IAEA isotopic reference materials with different certified isotopic compositions, as reported in Table 1.

### 2.5. Statistical Analysis

Statistical analyses were performed on replicate measurements (*n* = 3). For variables quantified in all three substrates (%C and δ^13^C), differences among GO, GO–ED, and GRAPHYMERE^®^ were evaluated by one-way analysis of variance (ANOVA), followed by Tukey’s post hoc test. For nitrogen-related parameters, pristine GO was excluded from statistical comparison because nitrogen was below the method detection limit; therefore, GO–ED and GRAPHYMERE^®^ were compared using Welch’s *t*-test. Differences were considered statistically significant at *p* < 0.05.

## 3. Results

**Figure 2:**
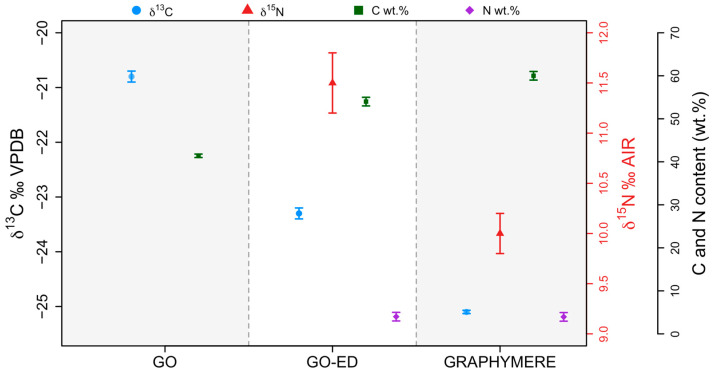
Carbon and nitrogen elemental composition (%C and %N) together with δ^13^C and δ^15^N values for GO, GO-ED, and GRAPHYMERE^®^. Error bars represent the standard deviation calculated on repeated measurement (*n* = 3).

Elemental and isotopic analysis results are reported in Figure 2. Pristine graphene oxide exhibited a carbon content of 41.4 ± 0.4 wt% and a nitrogen content below the method detection limit (0.5%) consistent with a highly oxidized carbon framework. Following functionalization with hexamethylenediamine, the carbon content increased to 54 ± 1 wt%, accompanied by the appearance of nitrogen (4.0 ± 0.1 wt%), indicating the introduction of nitrogen-containing functional groups. The final MMA-modified substrate (GRAPHYMERE^®^) showed a further increase in carbon content to 60 ± 1 wt%, while maintaining a nitrogen content of 3.95 ± 0.05 wt%.

These compositional changes are consistent with the progressive modification of the GO structure along the functionalization pathway shown in Figure 1, from pristine GO to the final MMA-modified material.

The carbon isotopic composition (δ^13^C) of the three substrates exhibited a clear trend towards more negative values with progressive modification of the GO structure (Figure 2). Pristine GO displayed a δ^13^C value of −20.8 ± 0.1‰. After amine functionalization, the δ^13^C value shifted to −23.3 ± 0.1‰ for GO–ED. The final MMA-modified substrate showed a further depletion in ^13^C, with a δ^13^C value of −25.11 ± 0.03‰.

A similar trend was observed for nitrogen isotopes. GO–ED exhibited a δ^15^N value of 11.5± 0.3‰, while GRAPHYMERE^®^ showed a slightly lower value of 10.0 ± 0.2‰. Pristine GO did not yield a measurable nitrogen isotopic signal due to a nitrogen concentration below the detection limit.

Statistical analysis confirmed significant differences in carbon content among the three substrates (one-way ANOVA, F(2,6) = 362.74, *p* < 0.001), with Tukey’s post hoc test indicating significant differences between all pairwise comparisons. Similarly, δ^13^C values differed significantly among GO, GO–ED, and GRAPHYMERE^®^ (one-way ANOVA, F(2,6) = 1807.48, *p* < 0.001), with all pairwise comparisons significant by Tukey’s test.

For nitrogen-related parameters, pristine GO was excluded from statistical comparison because nitrogen was below the method detection limit. Nitrogen content did not differ significantly between GO–ED and GRAPHYMERE^®^ (Welch’s *t*-test, *p* = 0.635), whereas δ^15^N values showed a significant difference between the two nitrogen-containing substrates (Welch’s *t*-test, *p* = 0.0019).

## 4. Discussion

The chemical modification of graphene oxide is commonly investigated by spectroscopic techniques such as FTIR and Raman spectroscopy. While these methods provide valuable qualitative information on functional groups and structural disorder, their diagnostic capability can be limited in complex and highly functionalized systems. In graphene oxide, the coexistence of multiple oxygen-containing functionalities, residual defects, and structural heterogeneity often results in extensive band overlap, which may obscure subtle spectral features associated with specific chemical reactions.

In Ruggiero et al., FTIR and Raman analyses suggested the functionalization of GO with hexamethylenediamine and the subsequent reaction with methyl methacrylate [16]. However, the presence of intense and overlapping signals prevented the unambiguous identification of diagnostic bands, leaving open the possibility that some spectral changes could arise from physical adsorption or non-covalent interactions rather than effective chemical modification. The present study addresses this limitation by employing elemental and stable isotope analyses as independent and quantitative probes.

### 4.1. Elemental Analysis: Functionalization and Reproducibility

Elemental analysis provides direct information on the bulk chemical composition of graphene-based materials. The transition from pristine GO to amine-functionalized GO (GO–ED) is characterized by a substantial increase in carbon content and by the introduction of nitrogen, consistent with the incorporation of amine-containing moieties through reaction with hexamethylenediamine in agreement with previously reported elemental trends for amine-functionalized graphene oxide, where nitrogen incorporation and a concomitant increase in carbon content (or C/O ratio) typically reflect successful functionalization [28,29].

Notably, the nitrogen content associated with GO–ED was reproducible across independent synthesis batches prepared under identical experimental conditions. This observation indicates that the extent of amine functionalization is governed primarily by the availability of reactive sites on the graphene oxide framework rather than by stochastic adsorption effects [2,30,31]. Such reproducibility is consistent with a controlled and repeatable chemical modification under the investigated experimental conditions, although it cannot provide direct bond-level confirmation of covalent attachment.

Following reaction with methyl methacrylate, the carbon content further increased in the final material (GRAPHYMERE^®^), while the nitrogen content remained essentially unchanged. This behavior is consistent with the involvement of pre-existing amine functionalities in the second reaction step and suggests that the overall degree of nitrogen incorporation approaches a saturation limit after amination [16,29,30]. The persistence of nitrogen after extensive washing and processing steps further excludes simple physical adsorption as the dominant mechanism.

### 4.2. Carbon Isotopic Composition as a Robust Indicator of Chemical Modification

Stable carbon isotope analysis provides a complementary and highly sensitive bulk probe of chemical modification. A clear and systematic shift in δ^13^C values is observed along the functionalization pathway, from −20.8 ± 0.1‰ for pristine GO to −23.3 ± 0.1‰ for GO–ED and −25.11 ± 0.03‰ for GRAPHYMERE^®^. These variations are well beyond analytical uncertainty.

The interpretation of the carbon isotope trend can be framed within a bulk isotope mass-balance approach [32,33]. For a material receiving carbon contributions from different pools, the isotope ratio of the final product can be approximated as shown in Equation (3):

(3) $$R_{product}=\frac{\sum_{i=1}^{k}R_{i}\cdot n_{i}}{\sum_{i=1}^{k}n_{i}}$$

where R_i_ is the carbon isotope ratio of the i-th carbon pool and n_i_ is its carbon contribution to the final material. In delta notation, and for small isotopic differences, this relationship can be expressed approximately as in Equation (4):

(4) $${\delta^{13}C}_{product}\approx \sum_{i=1}^{k}{\delta^{13}C}_{i}\cdot f_{i}$$

where f_i_ is the fractional carbon contribution of each pool. Within this framework, the observed monotonic δ^13^C shift is consistent with a progressive change in the bulk carbon composition of the material as new carbon-containing reagents are introduced during functionalization. Since the δ^13^C values of the individual reagents and removed fractions were not independently measured, this model is used here as a qualitative interpretive framework rather than as a quantitative source apportionment.

Several processes may contribute to this δ^13^C evolution. The primary contribution is expected to arise from bulk isotopic mass-balance effects associated with the progressive incorporation of carbon-containing reagents during the functionalization sequence [16,32,33]. This interpretation is supported by the concomitant increase in carbon content and by nitrogen incorporation after amination. Since carbon atoms in the aliphatic diamine backbone are not the primary reactive centers during amination, large and localized carbon isotope fractionation is not expected [31,32]; consequently, the δ^13^C signal is dominated by compositional changes rather than by reaction-specific fractionation effects. Additional secondary effects, including possible deoxygenation/decarboxylation or washing-related selective removal of labile oxidized carbon fractions, may also influence the final δ^13^C value [33,34,35,36]. However, since the isotopic compositions of the individual reagents, removed fractions, and washing solutions were not independently measured, the relative contributions of these processes cannot be quantitatively resolved in the present study. Accordingly, the δ^13^C trend is interpreted as a bulk isotopic descriptor consistent with progressive chemical modification, rather than as a quantitative apportionment of individual reaction mechanisms.

Within the investigated functionalization sequence, the monotonic δ^13^C evolution was reproducible and consistent with progressive chemical modification. However, since the elemental composition and oxidation degree of GO may vary with synthesis route, purification procedure, and commercial batch, its broader robustness as a bulk descriptor should be verified using multiple independently characterized GO lots.

### 4.3. Nitrogen Isotopes and Localized Fractionation Effects

Nitrogen isotope analysis further complements the compositional data. The δ^15^N values measured for GO–ED (11.5 ± 0.3‰) and GRAPHYMERE^®^ (10.0 ± 0.2‰) show a modest difference that can be reasonably attributed to changes in the chemical environment of nitrogen atoms during the second reaction step and to minor batch-dependent fractionation effects.

Unlike carbon isotopes, nitrogen isotopic composition can be sensitive to local bonding environments and reaction mechanisms involving nitrogen-bearing functional groups, including changes in protonation or coordination state [37]. Therefore, small variations in δ^15^N between samples are expected and do not undermine the interpretation of effective functionalization. Instead, the combination of a reproducible nitrogen yield and modest, chemically plausible δ^15^N variations supports a consistent and controlled reaction pathway [32].

### 4.4. Possible Deoxygenation Effects

Oxygen was not directly quantified in GO–ED and GRAPHYMERE^®^; therefore, the C/O ratio and the degree of deoxygenation cannot be quantitatively determined from the present dataset. The supplier-reported oxygen content of pristine GO confirms the highly oxygenated nature of the starting material, but no direct information on the oxygen content of the functionalized derivatives was obtained. Accordingly, deoxygenation or reduction-like processes during functionalization are discussed only as literature-supported possible contributions to the observed elemental and isotopic trends, not as experimentally demonstrated mechanisms in this work [28,31,33,34,35,36,38].

While deoxygenation-related processes may contribute to the carbon isotope signature, the monotonic δ^13^C trend is more consistently explained by progressive incorporation of external organic carbon pools, provided that large selective carbon losses from the GO framework did not occur [32,33].

Previous studies have shown that washing or base-washing procedures can remove oxidized carbonaceous debris from graphene oxide and modify the composition of the residual solid [36]. Therefore, in addition to possible deoxygenation or reduction-like processes, washing-related removal of labile oxidized fractions may also contribute to changes in the elemental and isotopic composition of GO-derived materials.

### 4.5. Complementarity of Analytical Approaches

Overall, the combined use of elemental and stable isotope analyses provides complementary bulk information on the sequential functionalization of GO to GRAPHYMERE^®^. EA–IRMS is not proposed here as a replacement for established characterization techniques such as XPS, TGA, solid-state NMR, FT-IR, or Raman spectroscopy, which provide surface-sensitive, thermal, structural, or vibrational information [2,16,30,31,34,35,36]. Elemental mapping by EDS/TEM may additionally provide spatially resolved information on elemental distribution, but not direct bond-specific confirmation [28]. By contrast, EA–IRMS combines bulk elemental composition with stable isotope signatures, allowing the functionalization sequence to be followed through elemental incorporation and isotopic mass-balance descriptors. Its value for this class of GO derivatives therefore lies in monitoring bulk chemical consistency and distinguishing the starting material, intermediate, and final derivative through integrated elemental–isotopic signatures, rather than in providing bond-specific structural confirmation.

### 4.6. Prospects for Dental-Material Research

The present study provides complementary analytical evidence consistent with the stepwise modification of GO into GO–ED and GRAPHYMERE^®^. Elemental composition and stable-isotope signatures allowed the three substrates to be differentiated at the bulk level the three substrates and complemented the previously reported FT-IR and Raman characterization.

These findings should not be interpreted as evidence of improved dental-material performance. Chemical functionalization does not necessarily ensure adequate dispersion, effective copolymerization, improved mechanical behavior, aging resistance, bio-compatibility, or antimicrobial activity.

The dental relevance of GRAPHYMERE^®^ therefore remains prospective. Future studies should incorporate defined concentrations into specific acrylic or methacrylate formulations and evaluate polymerization, dispersion, mechanical properties, aging, cytocompatibility, and biofilm-related outcomes.

Within these limits, EA–IRMS and elemental analysis are proposed as complementary methods for precursor and batch characterization, not as substitutes for formulation-level physicochemical, mechanical, or biological testing.

### 4.7. Limitations

Some limitations of the present study should be acknowledged. Oxygen content after functionalization was not directly quantified, and changes in oxygenated functionalities are inferred indirectly from elemental composition trends and from the certified composition of the pristine graphene oxide. Chemical processing of GO may induce partial deoxygenation or reduction-like effects, and the possible loss of carboxyl-derived carbon (i.e., decarboxylation) cannot be fully excluded. However, studies on the thermal reduction and deoxygenation of graphene oxide indicate that these processes primarily affect oxygen-containing functionalities, C/O ratio, and electronic structure [34,35,36]. Since the present study did not directly quantify oxygen after functionalization or monitor isotopic changes during isolated deoxygenation steps, a minor contribution of deoxygenation or decarboxylation to the observed δ^13^C trend cannot be excluded. Accordingly, any contribution from deoxygenation or decarboxylation is expected to be secondary with respect to the dominant mass-balance effect arising from the progressive incorporation of organic carbon during functionalization. Minor batch-dependent effects may influence nitrogen isotopic composition due to localized chemical environments. However, the overall carbon isotope trend remains systematic within the investigated functionalization sequence, although its quantitative interpretation should consider the possible secondary contributions discussed above.

Repeated washing steps may also affect the final carbon isotope composition if isotopically distinct labile fractions are selectively removed from the GO-derived solid [32,36]. Since neither the removed fractions nor the washing solutions were isotopically characterized, this contribution cannot be quantitatively resolved in the present study. Therefore, washing-related selective removal is acknowledged as a possible limitation in the interpretation of the δ^13^C trend, while the combined elemental and isotopic results remain consistent with progressive chemical modification during functionalization. Future studies using isotopically labelled reagents or isotope analysis of washing fractions could help distinguish incorporation effects from selective removal processes more directly.

In the present study, EA–IRMS was applied to obtain bulk C/N elemental data and δ^13^C/δ^15^N isotopic signatures and therefore does not provide direct bond-level confirmation of covalent attachment. Although the observed elemental and isotopic trends are consistent with the proposed functionalization pathway, complementary surface-sensitive or structural techniques such as XPS and solid-state NMR would be required to directly confirm the bonding environment [30,31].

In addition, oxygen content after functionalization and surface resistance or conductivity were not measured in this study. Direct oxygen determination, XPS, and electrophysical measurements should therefore be considered in future work to assess possible reduction or deoxygenation effects more directly [30,38].

Graphene oxide is a non-stoichiometric oxidized carbon material whose elemental composition and oxidation degree may depend on the synthesis route, purification procedure, and commercial batch [1,2]. Therefore, variability in the starting GO may affect the quantitative extent of functionalization. In this study, GO, GO–ED, and GRAPHYMERE^®^ were compared within the same functionalization workflow and under the same analytical conditions; however, broader batch-to-batch reproducibility should be assessed in future studies through batch-specific characterization of both the starting GO and the corresponding functionalized products. Finally, reproducible performance in polymer or dental materials will require formulation-specific studies correlating these analytical descriptors with mechanical, biological, aging, and application-dependent properties.

GRAPHYMERE^®^ was not incorporated into a dental resin; therefore, the present data cannot establish formulation-level mechanical, aging, biological, antimicrobial, or clinical performance.

## 5. Conclusions

Elemental and stable isotope analyses provided complementary quantitative evidence consistent with the stepwise chemical modification of graphene oxide into GO–ED and GRAPHYMERE^®^. Nitrogen incorporation after amination, the further increase in carbon content after methyl methacrylate modification, and the monotonic δ^13^C shift support progressive functionalization and help address some interpretive limitations of FT-IR/Raman spectra in chemically heterogeneous GO systems. By combining elemental composition and stable isotope signatures, this work strengthens the analytical characterization of GRAPHYMERE^®^ as a GO-based methacrylamide derivative. Accordingly, the present results should be interpreted as complementary evidence supporting the progressive chemical modification of GO during functionalization, rather than as standalone proof of covalent bond formation. Future studies should test this chemically characterized precursor in specific dental acrylic and methacrylate formulations under aging, mechanical, biological, and biofilm-relevant conditions.

## 6. Patents

The material and synthesis route discussed in this manuscript are covered by Italian Industrial Patent No. 102024000008704, “Materiale monomerico a base di ossido di grafene modificato con gruppo acrilammidico: destinazioni d’uso e relativi procedimenti di sintesi, produzione e lavorazione,” filed on 17 April 2024 and granted by the Italian Patent and Trademark Office on 23 April 2026. The named inventors are Gennaro Ruggiero, Davide Di Rosa, Giuseppe Caso, and Francesco Caso.

## Figures and Tables

**Table 1:** Selected IAEA reference materials and their matrices; isotopic values δ^13^C and δ^15^N (‰) with associated uncertainty are expressed relative to the international standards VPDB-LSVEC (carbon) and AIR (nitrogen).

IAEA Code	Matrix	δ Values (‰)
IAEA-C5	Subfossil wood (*Picea* sp.), “Two Creeks” (Wisconsin, USA)	δ^13^C: −25.49‰ ± 0.72‰
IAEA-C6	Cane sucrose (“ANU Sucrose”)	δ^13^C: −10.80‰ ± 0.47‰
IAEA-600	Pure caffeine (prepared in 2001)	δ^13^C _VPDB-LSVEC_: −27.771‰ ± 0.043‰ δ^15^N_AIR_: +1.02‰ ± 0.05‰
IAEA-NO-3	Potassium nitrate (KNO_3_), prepared 1983–1985	δ^15^N_AIR_: +4.7‰ ± 0.2‰
IAEA-N-2	Ammonium sulfate ((NH_4_)_2_SO_4_), prepared 1978–1983	δ^15^N_AIR_: +20.41‰ ± 0.12‰

## Data Availability

The original contributions presented in this study are included in the article. Further inquiries can be directed to the corresponding authors.

## Abbreviations

The following abbreviations are used in this manuscript:

GO	Graphene Oxide
ED	1,6-hexanediamine
MMA	Methyl Methacrylate
PMMA	Polymethylmethacrylate
FT-IR	Fourier-Transform Infrared spectroscopy
EA-IRMS	Elemental Analyzer–Isotope Ratio Mass Spectrometry
VPDB	Vienna Pee Dee Belemnite
LSVEC	Lithium carbonate prepared by H. J. Svec
NMR	Nuclear Magnetic Resonance
XPS	X-ray Photoelectron Spectroscopy
TGA	Thermogravimetric Analysis
EDS/TEM	Energy-Dispersive X-ray Spectroscopy coupled with Transmission Electron Microscopy
